# FHR vs. PFNA for femoral neck basicervical fractures in elderly patients 60 years or older: a cost-effectiveness analysis from hospitals in western China under the background of medical insurance

**DOI:** 10.3389/fsurg.2026.1723797

**Published:** 2026-03-17

**Authors:** Mingliang He, Yuhao Yan, Xuanze Liu, Guoqing Xiao

**Affiliations:** Department of Orthopaedic Surgery, The Second Affiliated Hospital of Chengdu Medical College (China National Nuclear Corporation 416 Hospital), Chengdu, Sichuan, China

**Keywords:** cost-effectiveness analysis, elderly adults, femoral neck basicervical fracture, FHR, PFNA, QALY

## Abstract

**Objective:**

Femoral neck basicervical fractures are an unstable subtype with high surgical failure risk. This study compares the cost-effectiveness of PFNA and FHR in elderly patients (60 years or older) to guide rational treatment amid aging and limited medical resources.

**Methods:**

A retrospective study included 76 patients (May 2022–May 2025) who underwent PFNA or FHR. 1:1 PSM balanced baseline characteristics (age, gender, pre-injury FRS, Garden classification, comminution, bone quality, AO/OTA subtype). Total hospital costs were collected to calculate CER and QALY-based ICER. FRS and EQ-5D-5L scales evaluated function and QALYs, with sensitivity analyses verifying robustness.

**Results:**

After PSM, 28 matched pairs were analyzed. PFNA had a lower CER (751.74 vs. 874.60 yuan/point, *p* = 0.002) and QALY-based CER (72,875.25 vs. 82,761.68 yuan/QALY). FHR required an additional 328,318.00 yuan/QALY (95% CI: 286,542.30–370,093.70), exceeding China's WTP threshold (85,698–257,094 yuan/QALY) but acceptable for specific subgroups. Both achieved favorable outcomes; FHR had faster early functional recovery.

**Conclusion:**

For elderly adults 60 years or older with femoral neck basicervical fractures, both PFNA and FHR can achieve favorable clinical outcomes. PFNA is more cost-effective overall, while FHR offers faster early functional recovery. Surgical decision-making should balance these factors, along with patient-specific characteristics. For patients prioritizing early independent ambulation and reducing caregiver burden, FHR is a suitable choice; for those focusing on cost-effectiveness and long-term functional recovery, PFNA is preferred. Future prospective multicenter studies with longer follow-up (5 years or longer) are needed to evaluate long-term revision costs and further validate these findings, while accounting for indirect costs and minimizing selection bias.

## Introduction

Hip fractures are among the most common and clinically serious fractures in the elderly, associated with a high risk of surgical failure (1). Approximately 10 million hip fractures occur worldwide each year (2). According to predictions based on the long short-term memory (LSTM) algorithm, by 2050, the age-standardized incidence rate of hip fractures among older adults globally is projected to reach 1,050 per 100,000 individuals (3). Femoral neck fractures can be divided into subcephalic, head-neck, transcervical, and basicervical types according to the location of the fracture line. Among them, basicervical fractures belong to a special subtype of femoral neck fractures. Because the fracture line is located outside the joint capsule, there is less damage to the medial and lateral femoral circumflex arteries and branches, and the fracture healing ability is strong. The risk of long-term avascular necrosis of the femoral head was significantly reduced compared with the first three fracture types, which was significantly different from the first three types of fractures. From the perspective of anatomy, the basicervical fracture of femoral neck belongs to a transitional type between transcervical fracture and femoral intertrochanteric fracture (4). From the perspective of biomechanics, basicervical fractures have higher shear stress and instability compared with other femoral neck fracture subtypes, and their biomechanical characteristics are closer to intertrochanteric fractures (5, 6). Therefore, in clinical treatment, some scholars prefer to treat the femoral neck basicervical fracture according to the treatment principle of femoral intertrochanteric fracture (7). For femoral neck basicervical fractures in the elderly, early surgical treatment can effectively reduce mortality and related complications compared with conservative treatment, which has become the mainstream concept (7–9). At present, surgical treatment schemes are mainly divided into two kinds: femoral head replacement and internal fixation. However, existing studies on the comparison of FHR and PFNA for femoral neck basicervical fractures are limited. Most prior investigations either focused on single surgical procedures or included mixed fracture sites (general femoral neck fractures or intertrochanteric fractures) rather than specifically targeting basicervical fractures (7–9). For example, Watson et al. (1) evaluated outcomes of cephalomedullary fixation (including PFNA) for basicervical fractures but did not compare with FHR. Lee et al. (10) analyzed risk factors for fixation failure in basicervical fractures treated with PFNA but lacked cost-effectiveness data relative to arthroplasty. Meanwhile, there are few reports on the cost-benefit comparison between FHR and PFNA in the treatment of femoral neck basicervical fractures. This research gap is critical because basicervical fractures have unique biomechanical and anatomical characteristics (higher instability than other femoral neck fractures but better healing potential than transcervical fractures) that may affect the cost-effectiveness of fixation vs. replacement.

Against the backdrop of population aging and tight medical resources, a reasonable treatment plan is particularly important. Through direct cost-effectiveness comparison of FHR and PFNA specifically for femoral neck basicervical fractures in the elderly, this study aims to fill the aforementioned research gap and provide evidence-based guidance for clinical decision-making.

## Material and methods

### Inclusion and exclusion criteria

For inpatient surgery patients who were mainly diagnosed with femoral neck basicervical fracture between May 2022 to May 2025, the following inclusion criteria were used: (1) Age ≥60 years old; (2) No other previous hip diseases; (3) Fracture site was fresh fracture (within 72 h after injury); (4) The surgical method was PFNA or FHR; Exclusion criteria: (1) Open fractures or combined with infection at the fracture site; (2) Old fractures; (3) Pathological fractures; (4) Combined with fractures in other parts requiring surgical treatment; (5) Cognitive impairment (mini mental state examination score; MMSE < 25); (6) The patient has symptoms such as hemiplegia; (7) Unable to cooperate with follow-up.

### Study design and patient allocation

This is a retrospective observational study. No randomization was performed, and surgical treatment was allocated based on predefined clinical criteria: FHR was preferred for patients with: (1) Garden III/IV displaced fractures (assessed via preoperative x-ray and CT); (2) Severe comminution of the fracture end (≥3 fragments); (3) Poor bone quality (T-score ≤ −2.5 on dual-energy x-ray absorptiometry); (4) High pre-injury activity level and expectations for rapid functional recovery. PFNA was selected for patients with: (1) Garden I/II minimally displaced fractures; (2) Minimal comminution; (3) Relative contraindications to arthroplasty (e.g., severe cardiopulmonary comorbidities, limited life expectancy); (4) Patient preference for preservation of the native femoral head. Femoral neck basicervical fractures are radiologically defined based on preoperative anteroposterior/lateral x-rays and CT scans: the fracture line is located at the base of the femoral neck, 1–2 cm below the femoral head-neck junction, extending transversely or slightly obliquely, involving the lateral cortex of the femoral neck without crossing the intertrochanteric line, which is consistent with the AO/OTA classification system 31B3 subtype.

### Propensity score matching (PSM)

To address potential confounding due to non-random allocation (age, gender, pre-injury functional status, degree of displacement, comminution, bone quality, AO/OTA fracture subtype), 1:1 PSM was implemented using SPSS 26.0. Logistic regression modeled propensity scores with covariates: age, gender and pre-injury FRS score, degree of displacement, comminution, bone quality, and AO/OTA fracture subtype. Nearest-neighbor matching without replacement was adopted with a caliper width of 0.2 standard deviations. Balance of baseline characteristics was primarily assessed via standardized mean differences (SMD), with SMD < 0.1 indicating adequate balance. After re-matching, all covariates showed no statistically significant differences between the two groups (all *p* > 0.05), and all SMD values were <0.1 (Table 1), confirming effective control of confounding factors. Additionally, pre-matching age imbalance (FHR group: 82.63 ± 7.89 years vs. PFNA group: 72.71 ± 6.54 years, *p* < 0.001) was effectively corrected post-matching (SMD = 0.08), minimizing the impact of age as a key geriatric confounding factor.

**Table 1:** Baseline and surgical characteristics (after PSM).

Indicators	FHR group (*n* = 28)	PFNA group (*n* = 28)	t/*χ*^2^	*P*	SMD
Gender (M/F)	7/21	6/22	0.089	0.766	0.04
Age (years)	77.56 ± 8.21	76.89 ± 7.93	0.281	0.782	0.08
Hospitalization Days	22.14 ± 12.87	19.86 ± 6.12	0.897	0.374	0.20
ICU Length of Stay (Days)	1.18 ± 4.02	0.43 ± 1.05	0.987	0.327	0.25
Operation duration (min)	117.50 ± 34.10	152.04 ± 78.24	2.216	0.034	0.53
Bleeding volume (mL)	278.57 ± 125.34	248.21 ± 190.76	0.678	0.501	0.18
Blood transfusion volume (mL)	361.79 ± 289.45	264.29 ± 325.61	1.012	0.316	0.31
Pre-injury FRS score	88.04 ± 2.71	87.32 ± 2.89	0.924	0.360	0.26
Pre-injury QALY	0.85 ± 0.07	0.84 ± 0.08	0.452	0.653	0.13
Displacement degree (Garden III-IV/I-II)	22/6	20/8	0.411	0.522	0.09
Comminution (severe/mild)	18/10	16/12	0.372	0.542	0.08
Bone quality (T-score ≤ −2.5/ > −2.5)	19/9	17/11	0.289	0.591	0.07
AO/OTA subtype (31B3.1/31B3.2, 31B3.3)	5/23	8/20	0.901	0.343	0.18

### Implant details

PFNA: WASTON Medical (CHN), proximal femoral nail anti-rotation (PFNA), length 170–210 mm, diameter 10–12 mm, 1 distal locking screw, proximal helical blade (diameter 11–13 mm) with endcap; surgical approach: lateral approach to the proximal femur; average cost 18,500 yuan.

FHR: AK Medical (CHN), cementless hemiarthroplasty, anatomical tapered stem with porous coating; head type: bipolar femoral head; head diameter 44–52 mm; offset: standard offset (40–45 mm); surgical approach: posterior lateral approach; rationale for cementless fixation: selected for patients with relatively preserved bone quality (T-score > −3.0) to avoid cement-related complications and enhance long-term osseointegration; average cost 25,800 yuan.

### Cost and outcome measures

#### Cost calculation methods

In China's medical system, the total cost of a patient's treatment is mainly composed of two parts: the medical insurance reimbursement part and the self-paid part. Different patients have different reimbursement ratios due to different types of medical insurance, so the final actual out-of-pocket expenses of patients are different. In this study, we only analyzed the total cost of treatment. The total cost includes pre-hospital treatment and inpatient treatment. Among them, (1) Pre-hospital treatment fees: including emergency examination fees, emergency registration fees, and pre-hospital emergency treatment fees (on-site fixation, medical monitoring and treatment costs during transportation) incurred for the diagnosis and treatment of the fracture from injury to admission. Pre-admission fees are provided by the patient upon admission. (2) Inpatient treatment expenses: obtained through the medical record system of our hospital. All cost data were adjusted to the 2022 price level using the Chinese Medical Consumer Price Index (CPI), with data from the annual medical and health CPI released by the National Bureau of Statistics of China (2022–2025). The adjustment calculation was based on 2022 as the base period, and the cost data of each year was multiplied by the ratio of CPI between the base period and the report period to complete the inflation adjustment to account for inflation during the study period (2022–2025). For international comparison, costs were converted to US dollars using the 2022 average exchange rate (1 USD = 6.722 CNY) and purchasing power parity (PPP, 1 USD = 3.99 CNY).

#### Outcome assessment tools and indicators

- FRS: Evaluates basic activities of daily living (44%), instrumental activities of daily living (23%), and walking ability (33%) (0–100 points, higher = better function) at baseline, 3, 6, and 12 months.
- EQ-5D-5L: Measures quality of life across five dimensions: mobility, self-care, usual activities, pain/discomfort, and anxiety/depression. Each dimension has 5 response levels (1 = no problem, 2 = slight problem, 3 = moderate problem, 4 = severe problem, 5 = extreme problem). The results at 3, 6, and 12 months postoperatively represent point utility values (i.e., utility at a specific time point). Cumulative quality-adjusted life years (QALYs) over the 12-month follow-up were calculated using the area-under-the-curve (AUC) method based on utility values at pre-injury (U0), 3 months (U3), 6 months (U6), and 12 months (U12) postoperatively. The corrected formula for total QALY calculation is: QALYtotal = [(U0 + U3)/2  ×  0.25] + [(U3 + U6)/2 × 0.25] + [(U6 + U12)/2 × 0.5]. This formula accounts for the 1-year time horizon (0–3 months: 0.25 years, 3–6 months: 0.25 years, 6–12 months: 0.5 years). Pre-injury utility values were derived from the EQ-5D-5L assessment completed by patients upon admission. QALYs were calculated using the Chinese utility value set for EQ-5D-5L (National Health Commission of China, 2021), which assigns a utility value to each health state defined by the scale.

#### Cost-effectiveness analysis

The cost-effectiveness ratio and incremental cost-effectiveness ratio of the two surgical methods were calculated separately. CER = Cost (total medical expenses)/FRS score, representing the cost required to obtain one additional unit of outcome. ICER was calculated exclusively based on cumulative QALYs over 12 months: ICER = [Cost (FHR total medical expenses)-Cost (PFNA total medical expenses)]/[QALY (FHR)-QALY (PFNA)], representing the additional cost required to obtain one additional QALY by choosing FHR over PFNA. The willingness-to-pay (WTP) threshold for comparison was based on China's 2022 per capita GDP (85,698 yuan), with 1–3 times per capita GDP per QALY (85,698–257,094 yuan/QALY) as the clinically acceptable range. Additionally, we conducted a qualitative description of the cost-effectiveness acceptability curve (CEAC) to reflect the probability of FHR being cost-effective at different WTP levels: at the lower bound of the WTP threshold (85,698 yuan/QALY), the probability of FHR being cost-effective is less than 20%; at the upper bound (257,094 yuan/QALY), the probability increases to 45%, indicating moderate uncertainty in FHR's cost-effectiveness relative to the standard threshold.

To enhance clinical interpretability, we additionally calculated the functional change amount (ΔFRS) as the difference between pre-injury FRS score and postoperative 12-month FRS score (ΔFRS = FRS score before the injury—FRS score at 12 months after the surgery). The corresponding cost-effectiveness ratio for functional change amount (CERΔFRS) was defined as CERΔFRS = Cost (total medical expenses)/ΔFRS, representing the cost required to achieve one additional unit of functional improvement after surgery.

### Statistical analysis

Statistical analyses were performed using SPSS 26.0 and Stata 17.0. Normality was tested via Shapiro–Wilk. Continuous data are presented as mean ± SD (independent samples *t*-test); categorical data as *n*(%) (*χ*² test). CER and ICER were calculated, with 95% confidence intervals (CIs) via bootstrapping. Sensitivity analysis adjusted implant costs, hospitalization days, and FRS scores by ±10%.

## Results

### Baseline characteristics after PSM

After PSM, 28 matched pairs (56 patients) were analyzed. Baseline characteristics, including age, gender and pre-injury FRS score, degree of displacement, comminution, bone quality, and AO/OTA fracture subtype were balanced between groups (all *p* > 0.05, all SMD < 0.1, Table 1). Power analysis (G*Power 3.1) confirmed the matched cohort of 56 patients (28 per group) achieved a statistical power of 87% (*α* = 0.05, d = 0.5), which exceeds the minimum required power of 80% (52 cases, 26 per group), confirming sufficient statistical power. Notably, pre-matching age imbalance (FHR group: 82.63 ± 7.89 years vs. PFNA group: 72.71 ± 6.54 years, *p* < 0.001) was effectively corrected post-matching (SMD = 0.08), minimizing age-related confounding.

### Surgical and clinical outcomes

All patients completed surgery without in-hospital mortality. The FHR group had significantly shorter operation duration (117.50 ± 34.10 vs. 152.04 ± 78.24 min, *p* = 0.034). Hospitalization days, bleeding volume, blood transfusion volume, and ICU stay showed no statistical differences (*p* > 0.05, Table 1).

Subgroup analysis of patients with stable fractures (Garden I-II, mild comminution) showed that the operative time of the FHR group (112.36 ± 28.45 min, *n* = 12) was significantly shorter than that of the PFNA group (148.52 ± 65.31 min, *n* = 14) (t = 2.135, *p* = 0.042). Other surgical indicators (bleeding volume, blood transfusion volume) in this subgroup showed no statistically significant differences between the two groups (all *p* > 0.05).

Perioperative complications were primarily lower extremity deep vein thrombosis (FHR: 12.5%; PFNA: 10.7%, *p* = 0.836). The average cost of patients with complications was 78,652.30 yuan, which was 23,415.60 yuan higher than that of patients without complications (*p* = 0.028). One FHR patient developed common peroneal nerve injury, resolving with conservative treatment. All PFNA patients achieved fracture healing at 1 year.

FHR group FRS scores were significantly higher at 3 (62.25 ± 1.70 vs. 59.68 ± 2.14, *p* < 0.01) and 6 months (67.52 ± 2.96 vs. 64.89 ± 2.44, *p* < 0.01), but not at 12 months (73.77 ± 3.07 vs. 72.64 ± 1.91, *p* = 0.052).EQ-5D-5L point utilities at 3, 6, and 12 months showed no statistically significant differences between groups (*p* > 0.05, Table 2).

**Table 2:** Postoperative functional outcomes and QALYs (after PSM).

Follow-up Time	FHR group (*n* = 28)	PFNA group (*n* = 28)	t/χ^2^	*P*
3-month FRS score	62.25 ± 1.70	59.68 ± 2.14	5.433	<0.01
6-month FRS score	67.52 ± 2.96	64.89 ± 2.44	4.181	<0.01
12-month FRS score	73.77 ± 3.07	72.64 ± 1.91	1.975	0.052
3-month EQ-5D-5L point utility	0.62 ± 0.10	0.58 ± 0.11	1.568	0.123
6-month EQ-5D-5L point utility	0.70 ± 0.11	0.67 ± 0.10	1.124	0.266
12-month EQ-5D-5L point utility	0.78 ± 0.12	0.75 ± 0.10	1.024	0.309
Total QALY (cumulative, 0–12 months)	0.70 ± 0.09	0.67 ± 0.08	1.342	0.184
3-month independent ambulation rate (%)	78.57 (22/28)	53.57 (15/28)	5.314	0.021
3-month full-time care rate (%)	42.86 (12/28)	67.86 (19/28)	4.528	0.033
12-month ADL independence rate (%)	82.14 (23/28)	78.57 (22/28)	0.152	0.689

* Point utility refers to the utility value at a specific follow-up time point.

Clinical outcome indicators related to functional status: At 3 months postoperatively, the proportion of patients achieving independent ambulation was 78.57% (22/28) in the FHR group and 53.57% (15/28) in the PFNA group (*χ*² = 5.314, *p* = 0.021); the proportion requiring full-time caregiver assistance was 42.86% (12/28) in the FHR group and 67.86% (19/28) in the PFNA group (*χ*² = 4.528, *p* = 0.033). At 12 months, the proportion of patients independent in activities of daily living was 82.14% (23/28) in the FHR group and 78.57% (22/28) in the PFNA group (*χ*² = 0.152, *p* = 0.689).

### Cost-effectiveness analysis

Total treatment costs were significantly lower in the PFNA group (54,658.69 ± 15,450.17 yuan; 8,133.73 ± 2,299.13 USD) than in the FHR group (64,554.11 ± 26,628.91 yuan; 9,606.27 ± 3,962.64 USD, *p* = 0.044). ICU costs were higher in the FHR group but not statistically significant (*p* = 0.075).

CER was 751.74 yuan/point (111.87 USD/point) for PFNA and 874.60 yuan/point (130.15 USD/point) for FHR (*p* = 0.002). CER*Δ*FRS (yuan/*Δ*FRS point) was 2,892.56 for PFNA and 3,421.79 for FHR (*p* = 0.003). QALY-based CER favored PFNA (72,878.25 vs. 82,761.68 yuan/QALY, *p* = 0.037). The QALY-based ICER showed that compared with the PFNA group, the FHR group required an additional 328,318.00 yuan (95% CI: 286,542.30–370,093.70) to obtain one additional QALY. This ICER exceeds China's commonly accepted WTP threshold (85,698–257,094 yuan/QALY, 1–3 times the 2022 per capita GDP of China) (Table 3).

**Table 3:** Cost and cost-effectiveness analysis (after PSM).

Indicators	FHR group (*n* = 28)	PFNA group (*n* = 28)	t/χ^2^	*P*
Total treatment costs (yuan)	64,554.11 ± 26,628.91	54,658.69 ± 15,450.17	2.050	0.044
Total treatment costs (USD)	9,606.27 ± 3,962.64	8,133.73 ± 2,299.13	2.050	0.044
ICU costs (yuan)	5,847.86 ± 16,947.71	1,314.77 ± 2,428.91	1.821	0.075
ICU costs (USD)	870.22 ± 2,521.98	195.65 ± 361.45	1.821	0.075
CER (yuan/point)	874.60	751.74	3.289	0.002
CER (USD/point)	130.15	111.87	3.289	0.002
CER (yuan/QALY)	82,761.68	72,878.25	2.146	0.037
CER (USD/QALY)	12,315.73	10,844.98	2.146	0.037
*Δ*FRS (pre-injury—12-month)	14.27 ± 3.52	14.68 ± 3.21	0.528	0.599
CERΔFRS (yuan/ΔFRS point)	3,421.79	2,892.56	3.215	0.003
CERΔFRS (USD/ΔFRS point)	509.04	430.31	3.215	0.003
ICER (yuan/QALY)	328,318.00	-	-	-
ICER (USD/QALY)	48,842.31	-	-	-
95% CI for CER (yuan/FRS point)	812.35–936.85	702.41–801.07	-	-
95% CI for CER (USD/FRS point)	120.89–139.41	104.53–119.21	-	-
95% CI for CER*Δ*FRS (yuan/*Δ*FRS point)	3,156.24–3,687.34	2,684.32–3,100.80	-	-
95% CI for CER*Δ*FRS (USD/*Δ*FRS point)	469.54–548.54	399.33–461.29	-	-
95% CI for ICER (yuan/QALY)	286,542.30–370,093.70	-	-	-
95% CI for ICER (USD/QALY)	42,627.54–55,057.08	-	-	-

## Discussion

The core focus of this study is the direct cost-effectiveness comparison between FHR and PFNA for elderly patients with femoral neck basicervical fractures. Our results show that while both procedures achieve favorable clinical outcomes at 1 year, they differ in short-term function and cost.

In terms of functional recovery, FHR provided significantly higher FRS scores at 3 and 6 months postoperatively, consistent with the advantage of arthroplasty in enabling early weight-bearing and rapid restoration of hip function (8). This is particularly valuable for elderly patients, as prolonged bed rest is associated with increased risks of pneumonia, deep vein thrombosis, and muscle atrophy. The clinical implications of the observed functional differences are substantial. The significantly higher FRS scores in the FHR group at 3 and 6 months directly translated into earlier independent ambulation and reduced caregiver burden. Early independent ambulation is particularly critical for elderly patients, as it reduces the risk of bed-related complications such as deep vein thrombosis, pneumonia, and muscle atrophy—complications that are associated with increased mortality and healthcare costs. For example, the 3-month independent ambulation rate was 25 percentage points higher in the FHR group, which may explain the similar complication rates between the two groups despite the more severe baseline fracture characteristics in the FHR group. However, the functional gap narrowed by 12 months, with no significant difference in FRS scores or QALYs between groups. This aligns with Lee et al.'s (10) finding that PFNA achieves comparable long-term function in basicervical fractures, albeit with slower early recovery. For PFNA, the gradual functional improvement may reflect the time required for fracture healing and osseointegration of the implant, whereas FHR bypasses the healing process by replacing the damaged femoral head.

In terms of cost, PFNA was significantly more cost-effective, with a lower CER (751.74 vs. 874.60 yuan/point) and total treatment cost (54,658 vs. 64,554 yuan). Despite FHR's QALY-based ICER exceeding the traditional WTP threshold, the uniqueness of elderly basicervical femoral neck fractures justifies further clinical interpretation. First, the significantly better early functional recovery of FHR reduces the risk of bed-related complications, which may lower subsequent healthcare costs and partially offset the incremental cost. Second, for patients with longer life expectancy and good pre-injury activity levels, long-term benefits (e.g., avoiding nonunion and secondary surgery) may improve FHR's cost-effectiveness in extended follow-up. Future studies with ≥5-year follow-up and inclusion of indirect costs are needed to re-evaluate its long-term cost-effectiveness. This balance between cost and short-term benefit is critical for resource allocation in China's healthcare system, where medical insurance coverage and healthcare budgets are constrained from a hospital perspective.

Few prior studies have directly compared the cost-effectiveness of FHR and PFNA for basicervical fractures. A retrospective study by Kweon et al. (11) reported similar clinical outcomes for cephalomedullary nailing (including PFNA) and hemiarthroplasty in basicervical fractures but did not include cost data. Our study extends this work by demonstrating that while FHR offers faster early recovery, PFNA is more cost-effective overall. This is consistent with general trends in femoral neck fracture management, where internal fixation is often preferred for minimally displaced fractures due to lower cost and preservation of the native hip, while arthroplasty is reserved for displaced fractures to avoid nonunion (7, 12).

However, it is worth noting that FHR (femoral head replacement) and THR (total hip replacement) differ in cost and outcomes in other healthcare systems. In Western countries, THR may be more cost-effective long-term due to lower revision rates, despite higher initial costs, as rehabilitation and nursing expenses constitute 70%–80% of total costs (9, 13). In China, however, FHR is often more cost-effective than THR because initial treatment costs account for 80%–90% of total costs, with low subsequent rehabilitation and nursing expenses (due to family caregiving and limited standardized rehabilitation) (14). This contextual difference highlights the importance of region-specific cost-effectiveness analyses.

Our study found that PFNA was more advantageous in terms of average hospital stay, bleeding volume, blood transfusion volume, and ICU length of stay, but the advantage was not significant. The FHR group was significantly less than the PFNA group in terms of operation duration, which had a significant advantage. The finding that FHR had a shorter operative time was further validated by subgroup analysis of stable fractures. Even in patients with similar fracture stability, FHR still showed a significant advantage in operative time, which may be attributed to the inherent procedural differences: FHR does not require precise fracture reduction, and the implant position is relatively fixed, reducing the time spent on intraoperative reduction and fluoroscopy. In contrast, PFNA requires accurate placement of the helical blade in the femoral head and confirmation of fracture reduction through multiple fluoroscopies, which inevitably extends the operative time. However, we acknowledge that patient selection (e.g., bone quality, comorbidities) may still have a minor impact on operative time, and future prospective studies should further control for these factors. This study found that the FRS score of the patients in the FHR group was significantly better than that in the PFNA group at the 3-month and 6-month follow-up, but there was no significant difference in the FRS score between the two groups at 12 months after the operation. It can be seen that in the short term after surgery, FHR patients can obtain better joint function, but there is no difference between the two in terms of long-term efficacy. Recent studies highlight surgical position and implant type's impact on outcomes. Studies (15–17) have shown that the intramedullary nail system represented by PFNA is significantly superior to lateral plate fixation and cannulated screw fixation in terms of biomechanical analysis. Yurteri et al. (18) demonstrated lateral decubitus position improves rotational alignment in intramedullary nailing. Nezir et al.'s research (19) shows that reducing intertrochanteric fractures of the femur under traction can achieve better Tip-apex distance. A 2024 Ann Med Res study (20) found PFNA and InterTan nails have comparable efficacy for unstable trochanteric fractures, with PFNA offering shorter operation times. These findings support surgical standardization and implant selection, informing future clinical practice.

This study has several important study limitations, many of which relate to inherent biases in retrospective observational design.

Selection Bias and Residual Confounding: Despite PSM balancing age, gender, and pre-injury FRS, unmeasured confounding factors may persist. Surgical allocation was based on clinical criteria (e.g., Garden classification, comminution, bone quality), which could introduce bias. For example, FHR was more likely for displaced/comminuted fractures (Garden III/IV), which are inherently more unstable. While PSM adjusts for observed covariates, residual confounding from unmeasured factors (e.g., surgeon experience, patient comorbidity severity) cannot be excluded. These unmeasured factors include comorbidity burden (e.g., severity of cardiovascular or respiratory diseases), frailty status (not assessed by standardized scales such as the Fried frailty phenotype), cognitive function (only MMSE < 25 was excluded, without detailed assessment of mild cognitive impairment), surgeon experience (variations in surgical proficiency between operators), perioperative management (e.g., thromboprophylaxis protocols, analgesia strategies), and rehabilitation intensity (differences in post-discharge rehabilitation participation). These factors may have influenced surgical outcomes and cost-effectiveness, and their potential impacts should be considered when interpreting the results.

Age Imbalance (Pre-matching Status and Post-matching Correction): Pre-PSM, the FHR group showed a tendency of aging [82.63 years vs. 72.71 years (PFNA group)], and this difference was statistically significant. Although we adjusted for age imbalance through PSM, age remains an important confounding factor in elderly fracture studies, as elderly patients may recover more slowly, have a higher incidence of complications, and have different costs.

Long-term Outcomes (Revision Risks and Unaccounted Costs): Our 1-year follow-up did not capture long-term revision risks. For FHR, revision to THR may be required due to acetabular wear, which would add significant costs. For PFNA, nonunion or avascular necrosis (though rare in basicervical fractures) could require revision to arthroplasty, increasing long-term costs. These unaccounted costs may alter the cost-effectiveness profile, with PFNA potentially becoming more cost-effective long-term if FHR revision rates are high.

Indirect Costs: We only included direct medical costs (pre-hospital and inpatient). Indirect costs (e.g., productivity loss, caregiver burden, transportation) were not measured, which may underestimate the true societal cost of each procedure.

Single-Center Limitation: The study was conducted at a single hospital in western China, limiting generalizability to other regions or healthcare systems. Patient demographics, treatment patterns, and cost structures may vary across China and internationally.

We should note that the choice of any surgical plan does not depend solely on treatment efficiency and cost, but also on the patient's physical condition and surgical indications. Although the treatment efficiency of FHR is low, it can effectively avoid the risks of fracture nonunion and avascular necrosis of the femoral head in elderly patients, and at the same time, it can allow patients to go down to the ground for early weight-bearing activities and reduce the related complications caused by bed rest. FHR may be more suitable for patients with long life expectancy, good physical condition, high pre-injury activity, and high requirements for joint co-function. PFNA is less traumatic, the patient recovers faster, and the perioperative risk is lower than that of FHR, but some joint function may be lost in the early and mid-term. At the same time, we need to pay attention to the complex and significantly displaced comminuted femoral neck basicervical fractures such as Garden III and Garden IV, PFNA may increase the difficulty of reduction, prolong the operation duration, increase the risk of intraoperative bleeding and anesthesia, etc. These findings provide practical guidance for clinical decision-making: FHR is preferred for patients with high pre-injury activity levels, good physical condition, and expectations for early mobilization, as the incremental cost is justified by the reduction in bed-related complications and caregiver burden. In contrast, PFNA is a more cost-effective option for patients with limited financial resources, lower activity expectations, or relative contraindications to arthroplasty, as it achieves similar long-term functional outcomes at a lower cost.

## Conclusion

For elderly adults aged 60 years or older with femoral neck basicervical fractures, both PFNA and FHR can achieve favorable clinical outcomes. PFNA is more cost-effective overall, while FHR offers faster early functional recovery. Surgical decision-making should balance these factors, along with patient-specific characteristics. For patients prioritizing early independent ambulation and reducing caregiver burden, FHR is a suitable choice; for those focusing on cost-effectiveness and long-term functional recovery, PFNA is preferred. Future prospective multicenter studies with longer follow-up (5 years or longer) are needed to evaluate long-term revision costs and further validate these findings, while accounting for indirect costs and minimizing selection bias.

## Data Availability

The raw data supporting the conclusions of this article will be made available by the authors, without undue reservation.
